# Collection of X-ray micro computed tomography images of shells of planktic foraminifera with curated taxonomy

**DOI:** 10.1038/s41597-023-02498-0

**Published:** 2023-10-05

**Authors:** Michael Siccha, Raphaël Morard, Julie Meilland, Shinya Iwasaki, Michal Kucera, Katsunori Kimoto

**Affiliations:** 1 MARUM Center for Marine Environmental Sciences, University of Bremen, Leobener Straße 8, Bremen, 28359 Germany; 2 Graduate School of Environmental Science, Hokkaido University, Sapporo, Japan; 3 Research Institute for Global Change, Japanese Agency for Marine-Earth Science and Technology, Yokosuka, Japan

**Keywords:** Marine biology, Palaeoceanography, Classification and taxonomy

## Abstract

Calcite shells of planktic foraminifera (Protista, Rhizaria) constitute a large portion of deep-sea sediments. The shells are constructed by sequential addition of partly overlapping chambers with diverse shapes, resulting in complex shell architectures, which are genetically fixed and diagnostic at the species level. The characterisation of the complete architecture requires three-dimensional imaging of the shell, including the partially or entirely covered juvenile chambers. Here we provide reconstructed x-ray micro computed tomography image stacks of 179 specimens of extant planktic foraminifera collected from plankton tows, sediment traps and surface sediments. The specimens have fully resolved and curated taxonomy and represent 43 of the currently recognised 48 holoplanktic species and subspecies. The image stacks form a basis for further applications, such as the characterisation of the architectural morphospace of the extant taxa, allowing studies of species functional ecology, calcification intensity and reconstructions of phylogenetic relationships.

## Background & Summary

Planktic foraminifera have a long service record as proxies for climatic reconstructions in marine geosciences. Their calcite shells are preserved in deep sea sediments and form an almost unrivalled archive for information on the state of the upper oceans during the organism’s life time. Particularly their shell chemistry, which covaries with ambient seawater conditions, has been in the focus of research. Interest in the biology of the living organisms has increased over recent years as it became apparent that a greater understanding of the living organism is required to increase the accuracy and precision of reconstructions based on their fossil remains.

X-ray micro-computed tomography is a non-destructive imaging technique that allows for the visualization and measurement of the internal structure of a sample. When applied to foraminifera it provides a way to image the internal and external morphology of foraminifera at high resolution, allowing for detailed analysis of the shell structure, chamber morphology and arrangement, and other morphological features. The method has been applied to investigate foramiferal shell architecture to characterise shell ontogeny^1–3^, shell dissolution in high resolution^4^, track ocean acidification^5^ and climatic changes^6^ and to support taxonomic divergence^7^.

The here described dataset of CT-scan image volumes of 179 specimens belonging to 43 the currently recognised 48 holoplanktic species and subspecies is the largest dataset published in this field so far. Including exclusively recent specimens from plankton tows, sediment traps and core top sediments it is intended primarily for morphological investigations of the extant taxa. Careful and exact segmentation of the shells allows for precise measurements of the size and shape of all chambers up to and including the primary one, the proloculus. A following segmentation of the inner chamber volumes will facilitate the investigation of the ontogeny (developmental trajectory) of the foraminiferal shells, potentially providing new insights into the phylogeny of planktic foraminifera. The distributed analysis of this dataset in the scientific community has the potential to contribute significantly to our understanding of the ecology, phylogeny and biomineralization of these important marine microorganisms.

## Methods

The scans were obtained at the three different institutions: the PACEA laboratory at the University of Bordeaux in Bordeaux, France; the Japanese Agency for Marine-Earth Science and Technology in Yokosuka, Japan and the MAPEX Center for Materials and Processes at the University of Bremen, Bremen, Germany.

Sample source selection for scans obtained at the universities of Bordeaux and Bremen followed the overarching goal of obtaining scans of the adult stages of all extant species of planktic foraminifera. We used three different types of source material, sediment, sediment trap and plankton tow, each material having its particular advantages and disadvantages for our purpose. Sediment is the most easily accessible type of material with samples available from all ocean basins covering the full diversity range of planktic foraminifera and containing almost exclusively adult specimens. It is however also the least desirable material in terms of contamination of the CT-scans by unwanted material such as sediment infill or smaller foraminifera. Plankton tow material, which is also abundantly available and covers all ocean basins, allows the collection of thin-walled species, e.g. *Hastigerina pelagica*, that are often not conserved in sediment samples. The specimens from plankton tows very often contain cytoplasm residue, even when selected from amongst the dead, visually clear specimens. This leads, in combination with their generally lower calcification intensity, in many cases to a considerably higher effort in the segmentation of the shell in the CT-scans. Material from sediment traps is ideal, generally exhibiting all the wanted characteristics such as adult morphology with high calcification intensity of the shell and low content of contaminants, but is only available from a limited number of locations.

Sample sources for the scans obtained at JAMSTEC were seafloor sediment samples bathed in calcite supersaturated bottom waters of the South Atlantic Ocean. Specimen selection focused on four abundant species to generate a dataset that could serve as a baseline for the evaluation of morphological variations and shell dissolution in seafloor sediment samples.

The sediment sample material used in this study underwent the standard sample preparation procedures for micropaleontological analysis, i.e. washing, drying and dry sieving. Similarly, sediment trap samples were prepared in standard fashion with multiple washing steps to remove the poisoning agent used during trap deployment from the samples. Afterwards sediment trap material was treated in the same way as sediment material. Samples from plankton tows were either live picked onto micropaleontological sample slides on board of the respective research vessel or picked from a thawed plankton sample in the laboratory. Specimens from plankton tows samples were exclusively cleaned with fresh water by manipulation with a picking brush. None of samples were cleaned ultrasonically, as we found this procedure entails  an unacceptable risk of damaging the previously carefully hand-picked specimens.

Regardless of the source material, individual specimens to be scanned were selected according to the criteria of exhibiting a species representative adult morphology and being undamaged and clean on the exterior as could be determined from observation with a binocular. Selected specimens for scanning were carefully placed on a layer of parafilm mounted on stubs (PACEA), individually glued with nail polish to a graphite rod (MAPEX) or mounted with tragacanth gum on a resin covered quartz glass stage at JAMSTEC.

A Phoenix V|tome|x S240 (*Baker Hughes, USA*) with settings of 80 kV, 180 µA without filter and a Zeiss Xradia 500 Versa (*Carl Zeiss AG, Oberkochen, Germany*) with settings of 80 kV, 88 µA and a Zeiss LE1 filter were employed at PACEA, the latter device and configuration was used exclusively for a singular scan (GE_GRU_2, *Globoturborotalita rubescens*). A ScanXmate-D160TSS105/11000 (*Comscantecno Co. Ltd., Kanagawa, Japan*) with settings of 80 kV, 10 µA with a 0.2 mm Al filter was used at JAMSTEC and a Zeiss Xradia 520 Versa (*Carl Zeiss AG, Oberkochen, Germany*) with settings of 80 kV, 86–88 µA without filter was employed at MAPEX.

**Fig. 1:**
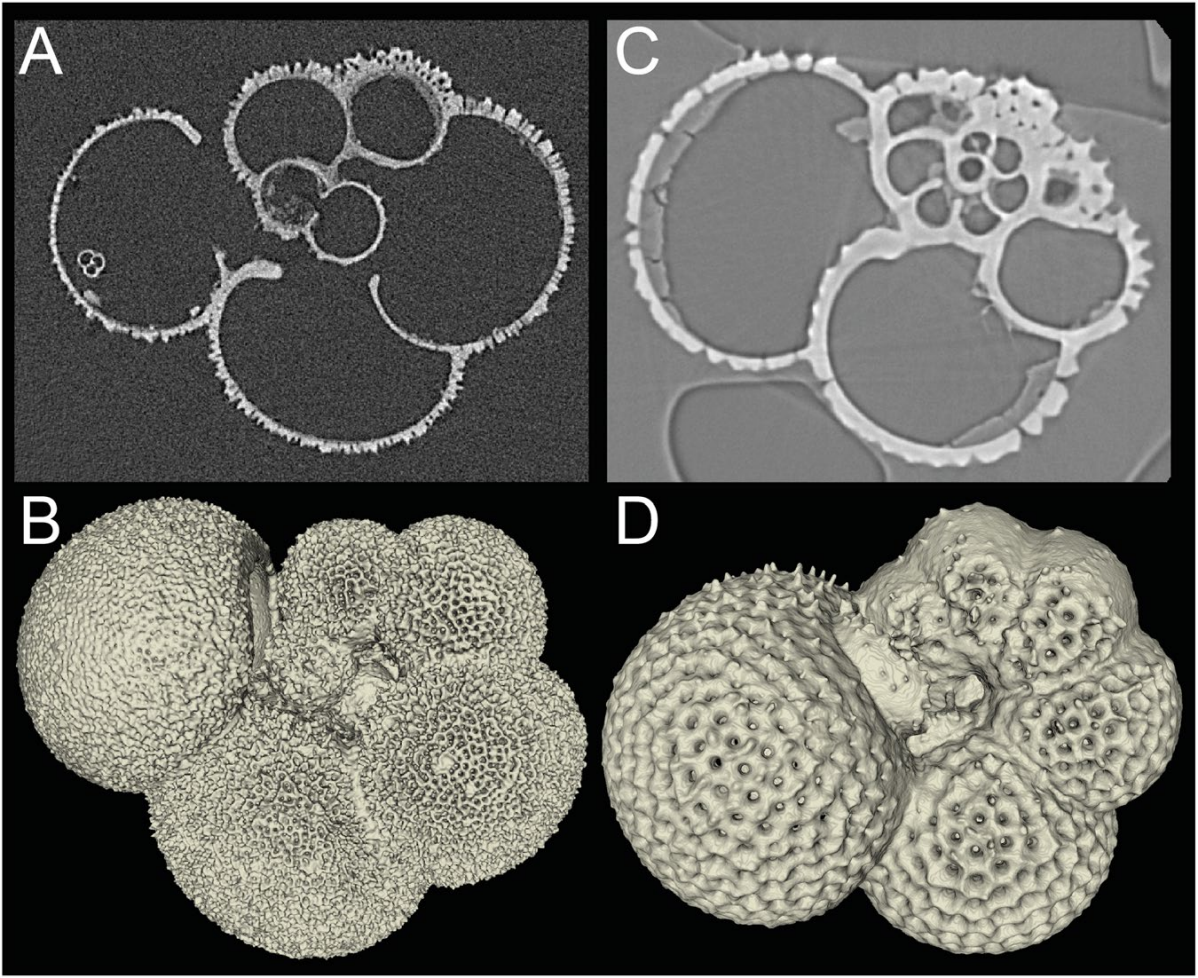
Exemplary cross-sections and shell segmentations. (**A**) cross section of data set GE_GSI_1, *Globigerinella siphonifera*. The specimen was obtained from a sediment sample. Residue of non-shell material attached to some of the interior chamber walls is visible. The final chamber (leftmost in the image) contains a small, juvenile foraminifera next to some other particles attached to the interior chamber wall. (**B**) Exemplary segmentation of the shell of data set GE_GSI_1. (**C**) cross section of data set GE_GHE_1, *Globigerinoides hexagonus*. The specimen was obtained from a plankton tow. Residue of dried cell plasma with homogeneous absorption values is visible in almost all chambers. (**D**) Exemplary segmentation of the shell of data set GE_GHE_1.

Correction of ring artifacts and reconstruction of the spatial information on the linear attenuation coefficient in the samples was done using the ZEISS Reconstructor software at PACEA and MAPEX or the ConeCTexpress software (Comscantecno Co., Ltd.) at JAMSTEC. Exemplary cross-section images and derived segmentation are shown in Figure 1.

## Data Records

Metadata for the 179 imaged specimens are given in supplementary table S1. The data publication^8^ in the PANGAEA data repository includes the same metadata table with download links for the individual image volumes in binary file format with file ending ‘raw’. The metadata include the fully resolved lowest taxonomic rank^9^, the data on specimen origin and sampling, oceanic region^10^ as well as the format information required to access the binary file correctly. The information on the data format is also included in the file name of each binary file itself. Particularities of the scanned specimen (aberrant shape) or the scan (missing or damaged chambers) are denoted in the comments of each entry.

The file naming follows the format:

$${\bf{S}}{\bf{c}}{\bf{h}}{\bf{e}}{\bf{m}}{\bf{e}}{\boldsymbol{:}}\,{\bf{A}}{\bf{B}}{\bf{C}}{\boldsymbol{\_}}{\bf{A}}{\bf{B}}{\bf{C}}{\boldsymbol{\_}}{\bf{123}}\,{\boldsymbol{[}}{\bf{123}}{\boldsymbol{\times }}{\bf{123}}{\boldsymbol{\times }}{\bf{123}}{\boldsymbol{-}}{\bf{123}}\,{\bf{u}}{\bf{m}}{\boldsymbol{-}}{\bf{A}}{\bf{B}}{\bf{C}}\,{\bf{A}}{\bf{B}}{\bf{C}}{\boldsymbol{]}}$$

ABC = Characters.

123 = A number.

Scheme elements not designated as “ABC” or “123” are fixed elements used as separators; this includes the spaces.

$${\bf{P}}{\bf{a}}{\bf{r}}{\bf{a}}{\bf{m}}{\bf{e}}{\bf{t}}{\bf{e}}{\bf{r}}{\bf{s}}{\boldsymbol{:}}\,{\bf{T}}{\bf{X}}{\boldsymbol{\_}}{\bf{G}}{\bf{S}}{\bf{S}}{\boldsymbol{\_}}{\bf{N}}{\bf{N}}\,{\boldsymbol{[}}{\bf{D}}{\bf{I}}{\bf{M}}{\bf{1}}{\boldsymbol{\times }}{\bf{D}}{\bf{I}}{\bf{M}}{\bf{2}}{\boldsymbol{\times }}{\bf{D}}{\bf{I}}{\bf{M}}{\bf{3}}{\boldsymbol{-}}{\bf{R}}{\bf{E}}{\bf{S}}\,{\bf{u}}{\bf{m}}{\boldsymbol{-}}{\bf{D}}{\bf{T}}\,{\bf{B}}{\bf{O}}{\boldsymbol{]}}$$

TX = Abbreviated suprageneric taxonomic group; e.g. GE for Globigerinidae.

GSS = Abbreviated genus and species name, e.g. CNI for *Candeina nitida*.

NN = Record number for specimen of the same species

DIM1, DIM2, DIM3 = dimensions of the volume in voxels.

RES = cubic resolution of the voxels in micrometers.

DT = datatype of the binary file elements, e.g. *ushort* for 16-bit unsigned integer.

BO = byte order of the binary file elements, BE for big-endian and LE for little endian.

$${\bf{E}}{\bf{x}}{\bf{a}}{\bf{m}}{\bf{p}}{\bf{l}}{\bf{e}}{\boldsymbol{:}}\,\text{'}{\bf{G}}{\bf{E}}{\boldsymbol{\_}}{\bf{T}}{\bf{S}}{\bf{A}}{\boldsymbol{\_}}{\bf{20}}\,[{\bf{460}}{\boldsymbol{\times }}{\bf{463}}{\boldsymbol{\times }}{\bf{350}}{\boldsymbol{-}}{\bf{1.0003}}\,{\bf{u}}{\bf{m}}{\boldsymbol{-}}{\bf{u}}{\bf{s}}{\bf{h}}{\bf{o}}{\bf{r}}{\bf{t}}\,{\bf{B}}{\bf{E}}]{\boldsymbol{.}}\,{\bf{r}}{\bf{a}}{\bf{w}}\text{'}$$

This file contains the raw data of the scan no. 20 of *Trilobatus sacculifer*, family Globigerinoidae, as a stream of 74,543,000 unsigned 16-Bit integers, without any file header, that need to be read into an array with a size of 460 by 463 by 350 elements in order to access the original CT-numbers of the voxels in 1.0003 µm cubic resolution.

Suprageneric taxonomic group, in most cases the family, is taken from the most recent taxonomy by Brummer&Kucera^9^; the main purpose for its inclusion here was the aim to generate short but unique species identifiers. The seemingly complex file naming was chosen to serve a better human readability and should allow accessing the data of an individual file with the information in the filename alone.

## Technical Validation

All data sets have been visually inspected and found to be free of any artefacts that could be attributed to the technical aspect of the data acquisition procedure. Damage to any specimen is indicated in the respective comments in the accompanying metadata. We publish the raw data without shell segmentations, since we are at the present moment unable to provide a complete, non-ambiguous methodology for shell segmentation that would be of sufficient quality to allow further analyses.

## Usage Notes

The provided data files are stored in RAW binary format. The information on image stack dimensions, voxel resolution, byte format and byte order are included in the file name.

Exemplary MATLAB code to import the RAW data of specimen GE_TSA_20 into a 3-dimensional array of unsigned 16-bit integers:

*      fid* = *fopen(‘GE_TSA_20 [460* × 463 × *350 - 1.0003 um - ushort BE].raw’,‘r’);*

*      V* = *fread(fid, ‘uint16* = *>uint16’,‘b’);*

*      V* = *reshape(V,460,463,350);*

Exemplary procedure to import the raw data of specimen CA_TIO_2 into ImageJ^11^ (https://imagej.nih.gov/ij/):

- *Select File-* > *Import-* > *Raw…from the main menu*
- *Choose file “CA_TIO_2 [340* × 331 × *267 - 0.4735 um - ushort BE].raw” in the file selection dialog*
- *Choose “16-bit Unsigned” for “Image type” – datatype “ushort”/ DT*
- *Enter “340” for “Width” – dimension 1*/*DIM1*
- *Enter “331” for “Height” – dimension 2*/*DIM2*
- *Retain “0” for “Offset to first image” – zero in all files as they include no header*
- *Enter “267” for “Number of images” – dimension 3*/*DIM3*
- *Retain “0” for “Gap between images”*
- *Uncheck “White is zero”*
- *Uncheck “Little-endian byte order” – for big-endian byte order*/*BE*
- *Uncheck “Open all files in folder”*
- *Uncheck “Use virtual stack”*
- *Confirm your settings with “OK”*

The segmentation of the data can be performed with various 3D image segmentation software suites. We found the program IKT-Snap^12^ (www.itksnap.org) convenient and powerful to obtain shell segmentations.

Experienced users might want to employ de-noising procedures to the raw data before further processing. We advise the users of the data that in almost every case a manual edit of any automatically generated shell segmentation is required for a satisfactory result. This can be attributed to varying calcification intensity of the shell obfuscating a global segmentation threshold or to the presence of particles or shell fragments in or on the shell that are by x-ray absorption alone indistinguishable from the calcareous foraminifera shell itself. In the few cases where only shell material is present the automatic segmentation procedures perform well.

The user is advised that due to use of several scanning devices at different laboratories with different instrumental settings and the lack of an accompanying calcite standard, a direct comparison of the CT-numbers for any type of shell density analysis is unfortunately not possible.

### Supplementary information

Supplementary Table 1: Image volume metadata

## Data Availability

No custom code has been used in the generation of the dataset.
